# Fermented Oats as a Novel Functional Food

**DOI:** 10.3390/nu15163521

**Published:** 2023-08-10

**Authors:** Richmond Djorgbenoo, Juanjuan Hu, Changling Hu, Shengmin Sang

**Affiliations:** Laboratory for Functional Foods and Human Health, Center for Excellence in Post-Harvest Technologies, North Carolina Research Campus, North Carolina Agricultural and Technical State University, Kannapolis, NC 28081, USA; rdjorgbenoo@aggies.ncat.edu (R.D.); jhu@aggies.ncat.edu (J.H.); chu@ncat.edu (C.H.)

**Keywords:** fermented oats, phytochemicals, bacteria, health benefits, functional food

## Abstract

Fermented oats are gaining popularity due to their nutritional value and the increasing consumer demand for health-conscious foods. These oats are believed to offer enhanced phytochemical and nutritional profiles compared to unfermented oats. The increased nutritional content of fermented oats is associated with various health benefits, including anti-inflammatory and antioxidant activities, which could potentially reduce the risk of chronic diseases. Further investigations are warranted to elucidate the nutritional benefits of fermented oats in human nutrition. This mini review provides a comprehensive overview of fermented oat products available on the market and the various production methods employed for fermenting oats. Furthermore, this review investigates how fermentation affects the chemical composition and biological functions of oats. Additionally, this manuscript presents some future perspectives on fermented oat products by discussing potential research directions and opportunities for further development. The findings presented in this review contribute to the expanding body of knowledge on fermented oats as a promising functional food, paving the way for future studies and applications in the field of nutrition and health.

## 1. Introduction

Fermented foods are products made through microbial action and the enzymatic conversion of food components [1]. During fermentation, carbohydrates like sugars and starch are broken down by yeast, fungi, or bacteria into simpler metabolites, enhancing the digestibility and absorption of vitamins, minerals, and other nutrients, making them functional foods with numerous health benefits [2,3]. When selecting strains for food applications, safety is the primary consideration. The European Food Safety Authority (EFSA) introduced the qualified presumption of safety (QPS) in 2007, requiring strains to be generally recognized as safe (GRAS). Additionally, the strains should be resistant to bile and acid, able to colonize the human intestine and attach to human epithelial cells, and possess antibiotic properties [4]. They should also withstand the matrix’s physiochemical conditions for successful application in foods [5], with the ability to remain viable during processing and storage to guarantee extended product shelf life. While traditional fermented dairy foods are commonly used for probiotic delivery [6], there is increasing demand for non-dairy probiotic options due to lactose intolerance, milk allergy, and interest in low-cholesterol products [7]. Cereals, fruits, and vegetable-based probiotic fermented products have gained attention as alternatives to dairy-based ones. Prebiotic ingredients, such as complex carbohydrates that probiotics can selectively metabolize, stimulate the growth of probiotic bacteria, enhancing probiotic functionality and efficacy [8].

Oats (*Avena sativa* L.) are considered one of the healthiest foods worldwide and are associated with numerous health benefits, including reducing the risk of cardiovascular diseases, type 2 diabetes, gastrointestinal disorders, and cancer [9]. It has also been shown that oats can promote the growth of lactic acid bacteria [10]. The health-promoting compounds in oats include not only beta-glucan (β-glucan) but also a series of phytochemicals, such as C-type avenanthramides (C-type AVAs), A-type avenanthramides (A-type AVAs), triterpenoid saponins, steroidal saponins, phenolic acids, flavonoids, and vitamins [11].

The production of oat-based fermented beverages started over three decades ago as the demand for functional food increased. Oat cereals are an essential functional food because they function as a fermentable substrate for the growth of probiotic microorganisms [12]. This mini review aims to offer a concise overview of fermented oat products as novel functional foods, exploring current products on the market, different production methods, the effects of fermentation on oats’ chemical composition and bioactivities, and future perspectives for fermented oats.

## 2. Fermented Oat Products on the Market

**Figure 1:**
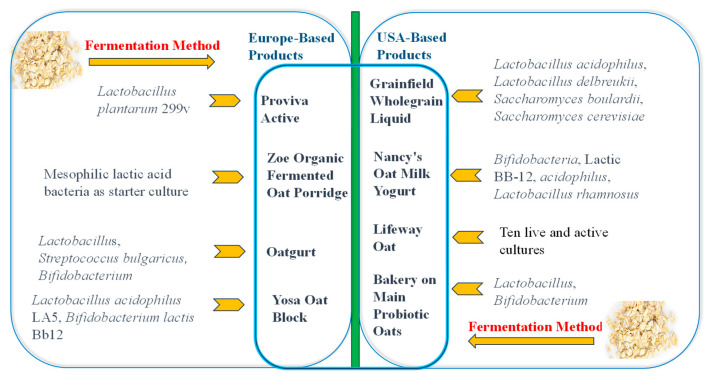
Some existing fermented oat products on the market with varieties of bacteria as the starter cultures.

Oat-based fermented products have been present on the European market for the past 30 years, fueled by increasing consumer interest in functional foods that offer nutritional and health benefits [13]. According to current knowledge, eight commercially available fermented oat-based products are found on the market, mainly in Europe and the United States (Figure 1).

One example is the Zoe organic fermented oat porridge, manufactured and marketed by UAB ProBIOduktai in Lithuania. This product is sold in a concentrated form and contains mesophilic lactic acid bacteria as its starter culture [14]. Oatly Group AB, a Swedish food company, produces and markets Oatgurt, a range of fermented oat products that contain active cultures such as *Lactobacillus, Streptococcus*, *Bulgaricus*, and *Bifidobacterium* [15]. Also, Probi AB and Skane Diary in Sweden produce Proviva Active using *Lactobacillus plantarum* 299v for fermentation [16,17]. Adavena M40 is another non-dairy fermented oat product from Sweden, with *Lactobacillus plantarum*, which consumers enjoy as a yogurt alternative [18]. Fazer, a Finnish company, produces Yosa Oat Block, a fermented oat product made with probiotic strains such as *Lactobacillus acidophilus* LA5 and *Bifidobacterium lactis* Bb12 [19].

In the United States, Nancy’s is a well-known brand that offers probiotic-rich dairy and non-dairy products. They produce oat milk yogurt made from fermented oats and live cultures such as *Bifidobacteria, lactis* BB-12, *Lactobacillus acidophilus*, and *Lactobacillus rhamnosus*, available in assorted flavors [20]. Grainfield Wholegrain Liquid is another product available in the USA, made from organic malted oats, rice, maize, pearl barley, alfalfa, linseeds, wheat, millet, and rye grain, and fermented using species such as *Lactobacillus acidophilus*, *Lactobacillus delbreukii*, *Saccharomyces boulardii*, and *Saccharomyces cerevisiae* [21]. Bakery on Main is a company known for producing probiotic oat products. Their probiotic oat products use specific starter cultures such as *Lactobacillus and Bifidobacterium* species. They offer a range of probiotic oat options in cups, including those that are fortified with either almond and vanilla; cranberry, apple, and almond; or walnut and banana [22]. Lifeway Oat, a USDA-certified organic oat beverage, is currently available on the market. It contains many non-dairy, vegan probiotic cultures, ranging from 25 to 30 billion colony-forming units (CFU). These cultures include *Lactobacillus casei*, *Lactobacillus plantarum*, *Bifidobacterium bifidum*, *Bifidobacterium animalis* subsp. lactis, *Bifidobacterium longum* subsp. longum, *Lactobacillus acidophilus*, *Lactobacillus paracasei*, *Lactobacillus rhamnosus*, *Lactobacillus lactis* subsp. lactis, and *Streptococcus thermophilus* [23]. Additionally, in 2018, the Quaker Oats company, owned by PepsiCo, filed a patent for fermenting oat flour and dairy together [24]. While this product has yet to be launched, if successful, it would be the first product in which grains and dairy are co-fermented simultaneously. The patent suggests that the prospective product may have unique metabolites that cannot be achieved through separate and sequential fermentation of grains and dairy, along with distinct sensory qualities. Goncerzewic and colleagues [25] studied another oat–banana beverage fermented using *Streptococcus thermophilus* TKM3KKP 2030p.

## 3. Methods Employed to Produce Fermented Oats

The final state of fermented oat beverages is influenced by various factors, including the time of fermentation, temperature, medium concentration, initial pH, and strain of microorganisms used, which affect the taste, color, and stability, as well as the levels of free phenolic compounds and reducing sugars [26]. The selection of appropriate fermentation conditions is crucial in the production of these beverages and greatly impacts sensory acceptance by consumers [12]. During fermentation, enzyme activity generally reaches its peak at the optimal fermentation time before declining [27]. Studies have shown that the highest number of living bacteria during oat fermentation occurs within the first 16 to 18 h, which correlates positively with the acidifying activities of the sample [28].

On the other hand, fermentation temperature affects strain growth and ultimately influences the flavor of the product. The choice of fermentation temperature depends on the characteristics of the fermentation substrate and the specific fermenting strains used [29]. Azadeh Asadzadeh and colleagues [30] employed *Bifidobacterium and Lactobacillus* to create a functional probiotic beverage using oat bran fermented at 40 °C. Notably, the optimum growth temperature for *Bifidobacterium* ranges between 36–38 °C and 41–43 °C for human- and animal-origin strains, respectively [31]. Consequently, when determining the fermentation temperature, it is crucial to consider both the optimal growth temperature and the optimal fermentation temperature to ensure favorable outcomes.

Regarding the starter organisms, more bacteria than fungi and yeasts have been employed in investigating phenolic profiles and antioxidant activities. Depending on the aim of the studies, either bacteria, fungi, or yeasts have been used to increase the contents in total phenolics, flavonoids, gamma-aminobutyric acid (GABA), β-glucan, and dephytinization. To ensure that enough cells are ingested by the host for viability in the gastrointestinal tract, a minimum of 6 Log colony forming units per milliliter (CFU mL^−1^) needs to be present in food for it to be considered a probiotic [32].

### 3.1. Bacteria

Lactic acid bacteria (LAB) are commonly used to ferment oats (Figure 1) because they possess the capability to metabolize various nutrients like proteins, fats, and lactose. In the process, LAB also enhance the content of soluble dietary fiber and phytochemicals, including phenolic compounds. This process improves digestibility and enhances the bioactivity of oat nutrients. Additionally, this process also produces substances like hydrogen peroxide, bacteriocins, and organic acids (such as lactic, acetic, propionic, and butyric acids), which can inhibit the growth of detrimental bacteria and pathogens, thereby improving food safety and preservation [33]. In previous studies, the most commonly used LAB species for oat fermentation belonged to the *Lactobacillus* (*Lb*) genus, such as *Lb. rhamnosus* [10,34], *Lb. fermentum* [35], *Lb. delbrueckii* subsp. *lactis* [36], *Lb. casei* [10,34], *Lb. acidophilus* [10,34], *Lb. brevis* [36], *Lb. johnsonii* [35], *Lb. reuteri* [29,35], and *Lb. paracasei* [34].

Different *Lactococcus (Lc)* strains have also been isolated and applied in the fermentation of oats, such as *Lc lactis* [37], *Lc. Lactis* sub. [35], *Lc. Sp cremoris,* and *Lc. lactis biovar diacetylactis* [38]. Another group of strains have been used in oat fermentation that belong to the genera *Pediococcus* (*P): P. acidilatici* [38], *P. pentisaceus* [39], *P. pentosaceus* [40], and *P. cellicola* [26]. Other bacteria such as *Bifidobacterium* (*Bifidobacterium animalis* ssp. *lactis), Enterococcus mundtii*, *Leuconostoc mesenteroides*, *Bacillus amyloliquefaciens* [41], and *Streptococcus thermophilus* [29] have also been reported.

In a specific study by Hole and colleagues [35], the aim was to increase the levels of phenolic acids during oat groat fermentation. They screened 56 LAB strains for their ferulic acid esterase (FAE) activity, which helps release bound phenolic acids into free forms during fermentation. Eight strains were selected, including four with high FAE activity (*Lb. johnsonii* LA1, *Lb. acidophilus* LA-5, *Lb. reuteri* SD2112, and *Bifidobacterium animalis* ssp. *lactis* BB-12), three with intermediate activity *(Lb. fermentum* NCDO 1750, *Lb. plantarum/pentosus* AD2, and *Lb. plantarum* NC8), and a negative control with no measured activity (*Lb. plantarum* WCFS1). These strains were then used to ferment oat groat at 37 °C for 18 h. The fermentation of oats with three probiotic strains, namely *Lb. johnsonii* LA1, *Lb. reuteri* SD2112, and *Lb. acidophilus* LA-5, resulted in the highest levels of free phenolic acids. These strains led to significant increases in phenolic acid content, ranging from 4.13 to 109.42 μg g^−1^ dry matter (DM). A study by Zhang and colleagues [37] revealed the prebiotic capability of oats and oat bran toward two prospective probiotics, *Lb. delbrueckii* subsp. *Lactis* and *Lb. brevis*, at 37 °C for 2 days, and the growth of *Lb. brevis* was best promoted by oats.

Other bacteria, like *bacillus amyloliquefaciens*, were used in a mixed grain containing 10% oat for solid state fermentation (SSF) at 37 °C for 36 h. A huge number of amino acids was observed compared to using individual grains [41].

### 3.2. Fungi

Some filamentous fungi that are on the GRAS list have advantages in producing functional fermented foods compared with lactic acid bacteria. Filamentous fungi have attracted attention for their advantage of being able to produce enzymes, such as *β*-glucosidase, esterase, phytase and xylanase, which can hydrolyze insoluble-bound nutrients, release free nutrient substances during the fermentation process, and also degrade plant cell walls to enhance bioavailability [42,43]. The fungi that have been employed in the fermentation of oats are *Aspergillus oryzae* var. *effuses*, *Aspergillus oryzae, Aspergillus niger*, *Rhizopus oryzae* [44,45], *Monascus anka* [42,43], *Sanghuangporus sanghuang* [46] and *Pleurotus ostreatus* [47].

Cai and colleagues [44] used four different filamentous fungi (*Aspergillus oryzae* var. effuses, *Aspergillus oryzae, Rhizopus oryzae*, and *Aspergillus niger*) to ferment oats at 25 °C for three days, and the results showed that all of the fungi could increase the content of GABA, total phenolic acids (TPC), or flavonoids. Diverse starter organisms can yield varying chemical profiles in the fermentation process. For instance, the highest levels of GABA were observed in oat fermentation with *Aspergillus oryzae*, while *Rhizopus oryzae* fermentation resulted in the highest TPC [44,45]. Additionally, a significant amount of soluble phenolics, ranging from 0.13 to 3.27 mg of gallic acid equivalents (GAE)/g dry weight (DW), was produced in oats fermented with *Monascus anka* for 14 days at 30 °C [42,43]. Fermentation by *Pleurotus ostreatus* has demonstrated increased protein digestibility, TPC, and antioxidant activity in oats [47]. Furthermore, when comparing solid substrates fermented with *Sanghuangporus sanghuang* (SS) for 12 days at 25 °C, oats fermented with SS displayed the highest content of total bioactive compounds and antioxidant activity among the fermented samples, including seven different grains [46]. Moreover, Wu and colleagues demonstrated that the fungus *Rhizopus oryzae* co-cultured with the bacterium *Lb. plantarum* showed better growth performance in whole-grain oats than raw oats, as evidenced by an increase in live cell count to 9.70 log CFU/g after 72 h of solid-state fermentation [48].

### 3.3. Yeasts

Yeasts generally can utilize sugars and lipids as carbon sources, while LAB primarily utilize various sugars for carbon utilization. Grzegorz Dąbrowski and colleagues [49] outlined the advantages of fermenting oats with a yeast strain—*Yarrowia (Y) lipolytica* YLP 0001. The study found that *Y. lipolytica* YLP 0001 fermentation increased acidity, characterized by higher levels of dissociated acids (such as lactic, citric, and succinic acids) and undissociated acids (such as amino acids and fatty acids). *Y. lipolytica* was identified as a significant aroma producer, where all fermented oat beverages were characterized by enhanced shares of branched, odd-chain, and cyclic fatty acids, with an increase from 5 to 1547 mg/100 g of lipids.

Additionally, while some microorganisms cannot synthesize folate de novo and strictly rely on external sources [50], yeast strains may harbor the genes for pathways in folate biosynthesis. For instance, some yeast strains, such as *Saccharomyces cerevisiae* ALKO743, *Saccharomyces cerevisiae* CBS7764, and *Candida milleri* ABM4949, have been reported by Kariluoto and colleagues [51] to show a significant capacity for folate production. Furthermore, *Saccharomyces cerevisiae* has been utilized in a study aiming to improve fermented oats’ phenolic content, and antioxidant capacity, by Călinoiu and colleagues [52]. The investigations revealed that *S. cerevisiae* exhibited a viable cell count of 8.58 log ± 0.11 log CFU/g and increased the TPC by 112% after four days of fermentation in oat bran.

## 4. Effects of Fermentation on the Chemical Composition of Oats

**Figure 2:**
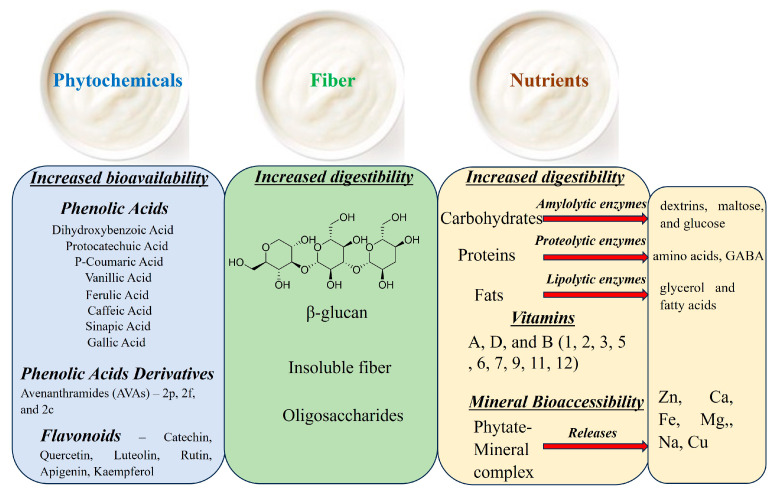
Impact of fermentation on the composition of oat phytochemicals, fiber, and nutrients.

Oats have a range of essential nutrients and non-nutritive phytochemicals that contribute to their nutritional value and potential health benefits. Fermentation can enhance the bioavailability and digestibility of these nutrients [47]. During fermentation, these microorganisms break down nutrients that are typically difficult for humans to digest, making them more accessible and bioavailable [53]. Additionally, microorganisms synthesize several essential B-complex vitamins and growth factors as they thrive in the fermentation medium. Furthermore, fermented oats can increase the contents of non-nutritive phytochemicals, which can enhance their potential health benefits. These phytochemicals, including phenolic acids and avenanthramides, and flavonoids have been linked to the antioxidative, anti-inflammatory, and potential anticancer effects of oats [11]. As a result, consuming fermented oats could provide a concentrated source of these nutrients and non-nutritive phytochemicals, contributing to a more nourishing diet (Figure 2).

### 4.1. Effect of Fermentation on Macronutrients in Oats

#### 4.1.1. Carbohydrates

Fermentation can lead to a decrease in the level of carbohydrates as well as some nondigestible poly- and oligosaccharides. The latter reduces side effects such as abdominal distension and flatulence [54]. The improvement in starch digestibility during fermentation can be attributed to the enzymatic properties of the fermenting microbiota, which actively break down starch and oligosaccharides (Figure 2). These enzymes are crucial in converting complex carbohydrates such as amylose and amylopectin into simpler forms, including dextrins, maltose, and glucose [55]. In one study by Carrie and colleagues [56], the carbohydrate content in raw oats was reported to be 66.0 g/100 g of grain. In another study by Das and colleagues [54], the carbohydrate content in fermented oats was reported to be 11.6 g/100 mL of water. To the best of our current knowledge, there is no direct comparison of carbohydrate levels in raw and fermented oats in a single study.

#### 4.1.2. Proteins

While it is anticipated that the quantity of oat protein may decrease after the fermentation process, it is important to note that there is no specific study that directly compares protein levels between raw and fermented oats. It has been reported that the fermentation of cereals by LAB increases free amino acids and their derivatives via proteolysis and/or by metabolic synthesis (Figure 2) [57,58]. Fermentation has been shown to improve the amino acid content (lysine, methionine, and tryptophan) of oats and other grains such as wheat, rice, and corn [59]. Furthermore, Cai and colleagues [44] observed a significant increase in the presence of GABA following a 72 h fermentation process involving filamentous fungi. GABA, an amino acid that serves as the primary inhibitory neurotransmitter in the central nervous system, exhibited approximately 6-, 8-, and 2-fold higher accumulation (330.9 μg g^−1^ oats, 435.2 μg g^−1^ oats, and 125.6 μg g^−1^ oats, respectively) compared to non-fermented oats (57.1 μg g^−1^ oats) when utilizing *A. oryzae* var. *effuses*, *A. oryzae*, and *R. oryzae*.

#### 4.1.3. Fats

Oats stand out among other cereals due to their elevated lipid content ranging from 2% to 13% [60]. The lipids found in oats play a crucial role in human nutrition as they are abundant in unsaturated fatty acids (e.g., linoleic acid and oleic acid) and essential fatty acids (e.g., myristic acid, palmitic acid, and stearic acid). The fat component constitutes approximately 5% to 9% of oat lipids [60]. During the fermentation process, lipolytic enzymes, such as lipases, facilitate the hydrolysis of fat into glycerol and various fatty acids (Figure 2), including lactic, acetic, butyric, formic, and propionic acids [61]. Consequently, the metabolic activities of microorganisms involved in oat fermentation can potentially reduce the fat content in oats. Although further research is required to directly compare the fat content of raw and fermented oats within the same study, Das and colleagues [54] reported a fat content of 6.79 g/100 mL of water in fermented oats.

### 4.2. Effect of Fermentation on Micronutrients in Oats

#### 4.2.1. Vitamins

Fermented oats possess high nutritional value, serving as a rich source of vitamins A, B, and D. Within the realm of vitamin synthesis, lactic acid bacteria demonstrate an ability to enhance the production of vitamins B2, B9, B11, and B12 [62]. In an investigation conducted by Das and colleagues [54], the fermentation of oats utilizing *Lactobacillus acidophilus* yielded substantial quantities of various B vitamins (including B1, B2, B3, B5, B6, B7, and B9), alongside the presence of vitamin E (Figure 2).

Folate, a vital vitamin within the B complex, plays a crucial role in maintaining human health, and its deficiency can lead to various health issues [13]. Kariluoto and colleagues [51] examined the folate content in fermented oats and barley rich in β-glucan. The study revealed that yeast cells generated 5-methyltetrahydrofolate and tetrahydrofolate, resulting in a net folate production of 120 ng/g in oats after 24 h of fermentation.

#### 4.2.2. Minerals

During the fermentation process of oats, the metabolic activities of microorganisms can lead to transformations and enhanced bioavailability of certain minerals. The specific minerals and synthesis mechanisms employed vary depending on the fermentation process and the microorganisms utilized [63]. Previous investigations focusing on lactic acid bacteria (LAB)-fermented oats have demonstrated notable quantities of essential minerals such as zinc, magnesium, iron, sodium, phosphorus, calcium, manganese, and copper [54].

Phytate, an anti-nutrient component, strongly binds to these minerals, reducing their bioavailability for absorption within the gastrointestinal tract [64]. However, evidence suggests that fermentation contributes to the hydrolysis of phytate, leading to a decrease in its levels and activity [65]. Fermentation creates favorable pH conditions for the enzymatic degradation of phytate, which forms complexes with polyvalent cations and proteins found in cereals (Figure 2). After 72 h of oat fermentation, Cai and colleagues [44] observed decreased levels of phytate in oats fermented with various fungi (reduced from 4.2 to 1.9 mg/g oats for *A. oryzae* var. *effuses*, from 4.2 to 1.6 mg/g oats for *A. oryzae* var., and from 4.2 to 2.4 mg/g oats for *R. oryzae*). This reduction in phytate content during fermentation can significantly enhance the solubility of iron, zinc, and calcium [66]. Further research is required to directly compare the mineral content of raw and fermented oats.

### 4.3. Effect of Fermentation on Fiber Content in Oats

Oats contain both soluble fiber in the form of β-glucan and insoluble fiber, along with other minor components like arabinoxylan and xyloglucan [67,68]. The substantial evidence supporting the beneficial role of oat β-glucans has prompted the US FDA to authorize health claims on oat products, attributing the reduction in cardiovascular disease risk to consuming at least 3 g per day of β-glucan [68]. However, despite the potential benefits of β-glucan, there have been conflicting findings in the literature regarding the impact of fermentation on β-glucan content, which is a crucial active component of oats. Gupta and colleagues [69] discovered that fermentation with lactic acid bacteria had no significant effect on the β-glucan content in oats. Fermented oat drinks containing 4%, 5.5%, and 7% oats exhibited average β-glucan concentrations ranging from 5.65% to 5.68%. Similarly, Bocchi and colleagues [70] observed that fermentation did not alter the oligosaccharide profile during the in vitro gastrointestinal digestion of fermented oat milk.

Conversely, Bernat and colleagues [71] reported a reduction of approximately 17% in the β-glucan content of fermented oat milk produced by bacteria. Mårtensson and colleagues [72] documented a 50% decrease in β-glucan levels following 16 h fermentation with *Bifidobacterium* DSM 20456. However, when fermented with *Lb. reuteri* ATCC 55730 and *Lb. acidophilus* DSM 20079 or with mixed cultures, no significant impact on β-glucan content was observed. Furthermore, preliminary studies have indicated that the absence of added sugar during fermentation may lead to a notable decrease in β-glucan content, potentially due to preferential utilization by *Lactobacilli* [73]. While some studies suggest that fermentation processes have an insignificant effect on β-glucan content in oats, others indicate reductions under specific conditions or with particular microbial strains. Further research is necessary to comprehend the factors influencing β-glucan content during fermentation and its implications for the nutritional properties of fermented oats.

### 4.4. Effect of Fermentation on Phytochemicals in Oats

**Figure 3:**
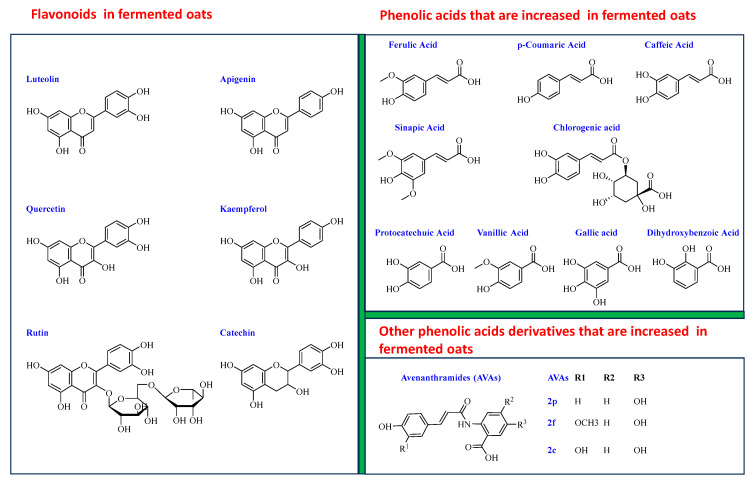
Structures of the major phytochemicals that are significantly elevated in fermented oats.

The health-promoting compounds in oats include not only β-glucan but also a series of phytochemicals, such as C-type avenanthramides (C-type AVAs), A-type avenanthramides (A-type AVAs), triterpenoid saponins, steroidal saponins, phenolic acids, and flavonoids [11]. Most of the phenolic acids and some of the flavonoids are present in their bond forms in oats. During fermentation, microorganisms produce enzymes, including α-amylase, glycoside hydrolase, *β*-glucosidases, cellulose- or xylan-degrading enzymes, and esterase. These enzymes play a role in releasing bound phenols present in the bran into free forms as shown in Figure 2 and Figure 3, enhancing their bioaccessibility and biological functions [44,74,75]. Fermentation also contributes to the breakdown of cereal cell walls by softening the kernel structure and liberating various bioactive compounds [45]. Studies have shown increased levels of total phenolic acids, individual phenolic acids, flavonoids, and C-type AVAs in fermented oats [45,76,77,78]. However, the effect of fermentation on other oat phytochemicals, such as A-type AVAs, steroid saponins, and triterpene saponins, remains unexplored [79,80,81]. It is important to note that the modifications of compounds in fermented oats can vary depending on factors such as extraction methods, the specific microorganism used, and the duration and temperature of fermentation.

#### 4.4.1. TPC

Several studies have investigated the impact of fermentation on the TPC of oats. Cai and colleagues [44] conducted a comparative study using three filamentous fungi (*Aspergillus oryzae* var. effuses, *Aspergillus oryzae*, and *Rhizopus oryzae*) for the solid-state fermentation of oats. The TPC increased over time, with the highest TPC observed in oats fermented with *Rhizopus oryzae* after 72 h. The TPC in these fermented oats was approximately twice as high as in unfermented oats. A similar study by Călinoiu and colleagues [52] used *Saccharomyces cerevisiae* as a starter culture for the solid-state fermentation of oat bran. The TPC reached its highest level on the fourth day, showing an 83% increase compared to unfermented oat bran.

Another study by Bei and colleagues [42] investigated oat fermentation using *Monascus anka* alone and in combination with *Bacillus subtilis*. Fermenting oats with *Monascus anka* for 12 days resulted in an 18-fold increase in TPC. When oats were fermented with a combination of *Monascus anka* and *Bacillus subtilis*, there was an even more significant increase, with TPC multiplying 23 times on the fourth day. These findings suggest that using different or multiple microorganisms may have a more pronounced effect on TPC than using a single microorganism.

However, a study by He and colleagues [82] reported a decrease in TPC during fermentation. Oats were fermented with *Lb. plantarum*, *Lb. acidophilus*, *Lb. casei*, *Lb. bulgaricus*, and *Streptococcus thermophilus* to compare the effects of fermentation on polyphenols. Except for the sample fermented with *Lb. casei*, all fermented samples showed a decreasing trend in TPC during the later stages of fermentation, especially after 12 h. After 48 h, only the oat sample fermented with *Lb. casei* showed a 3.14% increase in TPC. Additionally, within 24 h of fermentation, all five strains’ free polyphenols content (FPC) was higher than that of the control sample, while the bound polyphenol content (BPC) exhibited the opposite trend. This observation can be attributed to the microbial metabolic activity that converts BPC into FPC.

#### 4.4.2. Phenolic Acids

Phenolic acids are prominent constituents of oats, primarily concentrated in the bran. They can be classified into two major types: hydroxybenzoic acids and hydroxycinnamic acids. Oats contain hydroxybenzoic acids such as protocatechuic, syringic, vanillic, p-hydroxybenzoic, and gallic acids, as well as hydroxycinnamic acids, including ferulic, *p*-coumaric, o-coumaric, caffeic, and sinapic acids (Figure 3). 

Among these, ferulic acid is the predominant phenolic acid in oats, accounting for up to 90% of the total polyphenols [11]. Phenolic acids exist in soluble free acid forms, soluble conjugated forms esterified to sugars and low-molecular-mass compounds, and insoluble bound forms covalently attached to cell wall structural components. The conjugated and bound forms are the primary types found in cereal grains [42].

Several studies have investigated the individual phenolic acids in fermented oats. One study by Soycan and colleagues [11] examined eight phenolic compounds (gallic acid, chlorogenic acid, p-hydroxybenzoic acid, caffeic acid, vanillic acid, *p*-coumaric acid, sinapic acid, and ferulic acid) in free, conjugated, and bound phenolic fractions of unfermented and fermented oats. Their findings revealed significant increases in specific phenolic acids within the free phenolic fractions of fermented oats. Notably, gallic acid, chlorogenic acid, vanillic acid, and ferulic acid exhibited fold increases of 13.05, 47.31, 18.64, and 28.22, respectively. In the bound phenolic fractions, vanillic acid was undetectable in fermented oats, while *p*-coumaric acid, sinapic acid, and ferulic acid were the major components, showing fold increases of 44.19, 51.38, and 40.35, respectively. The conjugated phenolic content ranged from 20.60 ± 2.31 to 438.42 ± 5.66 mg/kg, with a nearly 100-fold increase observed in chlorogenic acid content.

In another study, Cai and colleagues [45] examined the phenolic composition of ethyl acetate (EA) subfractions in fermented oats using three different filamentous fungi. The EA subfraction of *A. oryzae* var. effuses-fermented oats showed approximately 3- and 9-times higher contents of caffeic acid and ferulic acid, respectively, compared to native oats. The EA subfraction of *A. oryzae*-fermented oats exhibited 2.7- and 5.5-times higher levels of caffeic acid and ferulic acid, respectively, than native oats. Both chlorogenic acid and *p*-coumaric acid increased more than two times in the *A. oryzae* var. *effuses* and *A. oryzae*-fermented oats. However, only caffeic acid and *p*-coumaric acid significantly increased the EA subfraction of *A. niger*-fermented oats.

Furthermore, Călinoiu and colleagues [52] investigated solid-state fermented oat bran using *S. cerevisiae* as a starter culture for a fermentation period of 0 to 6 days. They observed notable increases in specific phenolic acids during fermentation. On day 4, there was a significant increase of 68.81% in *p*-coumaric acid, followed by a 21.24% increase in ferulic acid and a 34.55% increase in dihydroxybenzoic acids. Sinapic acid exhibited the highest increase (+57.07%) on day two compared to the control.4.4.3. Flavonoids

Although typically found in cereals in small quantities, flavonoids play an essential role in their health benefits. As seen in Figure 3, the six major flavonoids reported in fermented oats are apigenin, luteolin, catechin, rutin, quercetin, and kaempferol [42,70]. Notably, these compounds are partly present in a bound form in oats, and can be hydrolyzed during the fermentation process. Cai and colleagues [45] compared the total flavonoid content in oats fermented with different fungi. The results showed significant increases in flavonoid values in oats fermented using *A. oryzae* var. *effuses*, *A. oryzae*, and *A. niger*. The concentrations of flavonoids in these fermented oats were 7893.1, 5749.9, and 5285.2 mg of rutin equivalents (RE) per 100 g of dry weight (DW), respectively, compared to the native oat content of 3714.8 mg of RE per 100 g of DW. Notably, luteolin, initially detected only in lesser amounts in native oats, increased after fermentation with all three fungi.

In another study by Bei and colleagues [42], four flavonoids (catechin, rutin, quercetin, and kaempferol) were identified and quantified in extracts of free, conjugated, and bound phenolic fractions from unfermented oats and *Monascus*-fermented oats using HPLC. Catechin was initially detected in low amounts only in the free and conjugated fractions, but after fermentation, its content in these two fractions significantly increased. Rutin and quercetin, initially present in low amounts or undetected in raw oats, were found in the conjugated fraction of fermented oats. The concentrations of rutin and quercetin in the conjugated fraction were 355.07 mg/kg and 42.35 mg/kg, respectively. This suggests that fermentation enhances the presence of these flavonoids in oats.

A study by He and colleagues [82] reported an increase in free flavonoids but a decrease in total flavonoid content during the fermentation of oats with five different strains of LAB (*Lb. plantarum*, *Lb. acidophilus*, *Lb. casei*, *Lb. bulgaricus*, and *S. thermophilus*). Following a 4 h fermentation period, the levels of free flavonoids increased in samples fermented using all four bacteria, except for *L. casei*, whereas the bound flavonoids decreased. Initially, *L. casei* displayed an opposite trend but eventually aligned with the same pattern as the other strains during the later stages of fermentation. Notably, at each fermentation time point, the content of free flavonoids in *L. casei* was significantly higher than that in the other strains, reaching 202.60 mg RE/L at 48 h. This represented a 20.47% increase relative to the unfermented samples.

#### 4.4.3. AVAs

Avenanthramides, distinctive phytochemicals found in oats, comprise an amide conjugate formed between anthranilic acid and hydroxycinnamic acids. There are three major types of C-type avenanthramides (AVAs): those derived from 5-hydroxyanthranilic acid with *p*-coumaric acid (referred to as 2p or A), ferulic acid (referred to as 2f or B), and caffeic acid (referred to as 2c or C) [83].

Two studies have investigated the impact of oat fermentation on the content of avenanthramides [45,52]. In one study involving the fermentation of oats with *S. cerevisiae*, a significant increase in avenanthramides (2f, 2p, and 2c) was observed on day 4, with respective increases of 48.45%, 42.10%, and 34.50% compared to unfermented oats [52]. Similarly, in oat samples fermented with three different fungi (*A. oryzae* var. effuses, *A. oryzae*, and *A. niger*), the contents of 2f, 2p, and 2c were also increased [45].

## 5. Biological Function of Fermented Oats

**Figure 4:**
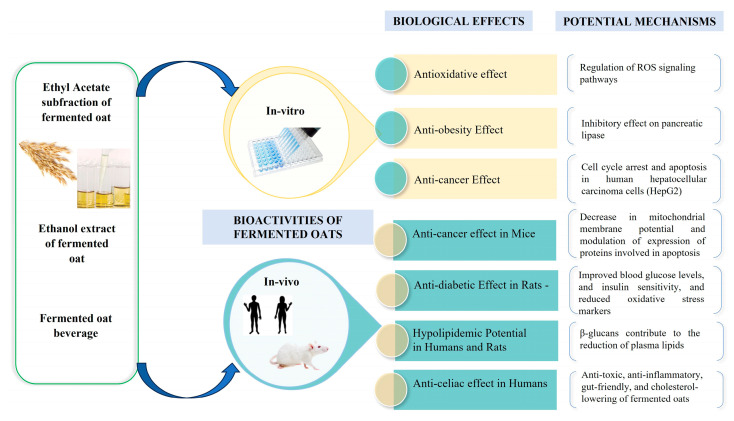
Biological functions of fermented oats.

Fermented oats are associated with a wide range of health benefits owing to their improved nutrient bioavailability and increased levels of beneficial compounds. These advantages encompass enhanced blood sugar regulation, assistance in weight management, lowered cholesterol levels contributing to improved cardiovascular health, a decreased risk of specific types of cancer, and therapeutic effects in celiac disease treatment (Figure 4).

### 5.1. Antidiabetic Effects of Fermented Oats

Gohari and colleagues [84] conducted a study to investigate the potential anti-diabetic and hypolipidemic effects of unfermented and fermented oat milk in alloxan-induced diabetic rats. The study employed forty-two male Albino rats as the animal model, inducing diabetes using alloxan. The rats were treated for four weeks and administered 5 mL of fermented oat milk (FOM) fortified with whey protein concentrate. The fermentation process involved specific strains, including *Lb. acidophilus*, *S. thermophilus*, and *Bifidobacterium bifidium*. Chemical composition analysis and the evaluation of various biomarkers were conducted to assess the impact of fermented oat milk. These biomarkers included the liver-to-body weight ratio, pancreas-to-body weight ratio, glucose levels, triglycerides (TG), low-density lipoprotein cholesterol (LDL-C), high-density lipoprotein cholesterol (HDL-C), and total cholesterol (TC), as well as the activity of liver enzymes such as alanine aminotransferase (ALT) and aspartate aminotransferase (AST). The findings demonstrated diverse effects across the evaluated biomarkers. The administration of FOM led to reduced glucose levels (28.3% reduction), improved lipid profiles characterized by lower TC (reduced from 155.33 ± 1.37 to 115.33 ± 0.71 mg/dL), TG (reduced from 89.33 ± 0.14 to 50.00 ± 0.31 mg/dL), and LDL-C (reduced from 76.00 ± 1.22 to 66.33 ± 0.54 mg/dL), and higher HDL-C levels (increased from 27.00 ± 0.71 to 45.33 ± 0.98 mg/dL). Additionally, it resulted in decreased liver enzyme activity and alterations in the composition of the intestinal microbiota, characterized by increased counts of *Lactobacilli and Bifidobacteria*. The study sheds light on the potential advantages associated with consuming fermented oat milk, particularly in ameliorating glucose control, enhancing lipid metabolism, promoting liver health, and influencing the composition of the intestinal microbiota, in rats with diabetes (Figure 4).

In another study, Alharbi and colleagues [85] investigated the effects of *Lactobacillus plantarum*-fermented oats and fermented oats supplemented with Sidr honey on type 2 diabetes in rats. Diabetes was induced by administering a solution of streptozotocin (STZ) in 0.1 M citrate buffer with a pH of 4.5 at a dosage of 45 mg kg^−1^ BW. After 72 h of fermentation, the honey-supplemented fermented oats showed more desirable effects on TPC (increased from 1.41 to 1.64 mg GAE g^−1^). The antioxidant capacity of the fermented oats was significantly increased, as indicated by the 2,2-diphenyl-1-picrylhydrazyl (DPPH) and 2,2′-azino-bis(3-ethylbenzothiazoline-6-sulfonic acid) (ABTS) measurements (increased from 5.12 to 7.15 µmol of Trolox Equivalent antioxidant capacity per gram (TE g^−1^) of DPPH and from 7.68 to 11.73 µmol of TE g^−1^ of ABTS). Oral administration of the fermented oat extract for six weeks effectively improved diabetic complications in rats, including blood glucose levels, lipidemia, liver and kidney functions, and oxidative stress. For instance, the administration of fermented oats demonstrated beneficial effects on blood glucose levels during the 0th, 3rd, and 6th weeks of treatment, with values of 308.5 ± 22.13 mg dL^−1^, 244.83 ± 28.82 mg dL^−1^, and 232.50 ± 15.24 mg dL^−1^, respectively. When fermented oats were supplemented with honey, the effects were even more pronounced, resulting in blood glucose levels of 320.83 ± 39 mg dL^−1^ for the 0th week, 225.17 ± 18.35 mg dL^−1^ for the 3rd week, and 211.50 ± 32.66 mg dL^−1^ for the 6th week The study demonstrated that the fermented oat extract, especially when supplemented with Sidr honey, exhibited significant antioxidative and ameliorative effects against type 2 diabetes. The presence of bioactive compounds such as TPC and GABA in the fermented oats and the synergistic effects of Sidr honey likely contributed to the observed benefits.

Furthermore, Algonaiman and colleagues [86] investigated the effects of fermented oats on diabetes. Fermented oats were prepared by fermenting whole oat flour with *Lactobacillus plantarum* for 72 h. Diabetes was induced in the rats via the intravenous administration of STZ dissolved in 0.1 M citrate buffer at a pH of 4.5. The dosage of STZ used for induction was 45 mg kg^−1^ BW. The fermented oat extract, LFOE, was administered orally to diabetic rats for six weeks. The results showed that fermented and unfermented oat extracts effectively reduced blood glucose levels. LFOE had slightly better effects, but both extracts significantly improved insulin sensitivity and reduced lipid levels. The fermentation time was crucial, as longer fermentation increased the viable *Lb. plantarum* count and the production of beneficial compounds like GABA and phenolic compounds. The fermentation process decreased β-glucan content but did not significantly affect the effectiveness of LFOE in improving hyperglycemia. Both extracts showed hepatoprotective and nephroprotective effects, improving liver enzymes and serum proteins. The fermentation process increased the extracts’ TPC and antioxidant capacity.

Overall, the existing studies suggest that fermented oats have significant antidiabetic, hypolipidemic, hepatoprotective, and antioxidant effects, which could be attributed to the production of bioactive compounds during fermentation. Further research is needed to identify the active components in fermented oats and understand the underlying mechanisms of these effects.

### 5.2. Anti-Obesity Effects of Fermented Oats

Studies have shown that pancreatic lipase plays a key role in splitting triglycerides into bioavailable glycerol and fatty acids in the gastrointestinal tract in humans [87]. Reducing triglycerides by inhibiting the digestion and absorption of dietary triglyceride could be an effective strategy to treat obesity [88]. In their study, Cai and colleagues [89] evaluated the inhibitory effects of nonfermented and fermented oats’ ethyl acetate (EA) subfractions on pancreatic lipase. They found that the fermented oats’ EA subfraction exhibited more potent lipase inhibition due to increased phenolic acid content from fungal fermentation. The study highlighted *p*-coumaric, caffeic, and ferulic acids as the primary compounds responsible for lipase inhibition (Figure 4). These phenolic acids showed concentration-dependent effects, with *p*-coumaric and ferulic acids displaying more potent inhibitory effects than caffeic acid. Synergistic interactions were observed at high concentrations (250 μg/mL) but additive effects occurred at low concentrations (50 μg/mL), suggesting that lower concentrations of these phenolic acids contribute additively to lipase inhibition. The study proposed fermented oats and polyphenol-rich foods as potential complementary therapies for obesity treatment. However, further research, particularly human studies, is needed to validate these findings in human subjects.

### 5.3. Anti-Cholesterol Effects of Fermented Oats

A positive association exists between elevated serum cholesterol levels and increased susceptibility to ischemic heart disease [90]. Numerous studies have demonstrated that foods rich in soluble dietary fiber can effectively reduce blood lipid levels by inhibiting the absorption of cholesterol and bile acids in the intestines [88]. Oats, known for their high concentration of soluble fiber, especially β-glucan [91], have been the subject of many investigations exploring the impact of oat products on serum cholesterol levels (Figure 4).

Mårtensson and colleagues [92] conducted a comparative study evaluating the effects of fermented oat-based products and fermented dairy yogurt on plasma lipid levels. The fermented oat-based products were derived from the liquid oat base Adavena M40, while the ropy oat-based product was formulated using the concentrated oat base Adavena G40. The two starting materials were all commercially acquired from Ceba Foods in Sweden. Adavena G40, containing native β-glucans from oats and microbial glucans from *Pediococcus damnosus* 2.6, resulted in a fermented product with a “ropy” characteristic (thick stringy texture). Human participants with moderately elevated cholesterol levels were divided into three groups: one consuming the fermented dairy product and the other two groups consuming different oat-based products. Fecal bacteria count and *Bifidobacterium* ssp. were measured to assess the prebiotic potential of the products. The group consuming the ropy oat-based product with a daily intake of 3.5 g of β-glucans showed a significant decrease in total cholesterol levels compared to the dairy-based product group. However, the other oat-based product with a daily intake of 3.0 g of β-glucans did not significantly affect plasma lipids. The concentration of β-glucans in the products influenced their effectiveness, and the presence of microbial β-glucans in the ropy oat-based product potentially contributed to lipid reduction. The study suggested that consuming approximately 3.5 g of native glucans daily through a fermented, ropy, oat-based product can effectively lower plasma lipid levels. Future research should investigate the physiological impact of isolated microbial β-glucans on plasma lipid reduction, and the fermented oat-based product shows potential as a prebiotic based on increased fecal *Bifidobacterium* ssp. counts.

### 5.4. Anti-Cancer Effects of Fermented Oats

Fermented oats, which have improved nutritional properties and are rich in polyphenols, were investigated by Zhang and colleagues [93] for their potential as anti-cancer drugs. The study examined the EA subfractions obtained from fermented oats prepared with *Rhizopus oryzae* 3.2751, which exhibited high polyphenol content. The EA subfractions demonstrated significant anti-cancer activity against four human cancer cell lines (MDA-MB-231, HepG2, HT-29, and HeLa) while showing minimal toxicity toward normal cells.

Male and female KM mice (5–6 weeks old) and female BALB/c nude mice (5–6 weeks old) were used in the animal model. The mice were intraperitoneally administered EA subfractions dissolved in corn oil at graded doses of 5, 10, 20, 40, and 60 mg/kg body weight daily for 24 days, with an injection volume of 10 mL/kg body weight. The control group received intraperitoneal injections of corn oil alone. In vivo experiments conducted on tumor-bearing mice revealed the inhibitory effects of the extracts on tumor growth without causing significant toxicity. The study further investigated the impact of the extracts on HepG2 cells (human hepatocellular carcinoma cells) to elucidate the underlying mechanisms. The extracts induced cell cycle arrest at the G2/M phase, triggering apoptosis in HepG2 cells. They also led to a reduction in mitochondrial membrane potential and modulated the expression of apoptosis-related proteins. Moreover, the extracts increased intracellular reactive oxygen species (ROS) levels and activated the ROS/JNK signaling pathway, contributing to the induction of apoptosis. These findings suggest that the anti-cancer effects of the fermented oat extracts involve multiple mechanisms, including cell cycle arrest, apoptosis, and the regulation of ROS signaling pathways (Figure 4).

### 5.5. Anti-Celiac Effects of Fermented Oats

Celiac disease (CD) is an immune-mediated condition triggered by gluten exposure in individuals with specific genetic predispositions [94]. Oats have been classified as gluten-free (GF) and can be included in the diet of most people with gluten intolerance, as per the Commission Implementing Regulation (EU) No. 828/2014. However, in some Southern European countries, oats were traditionally excluded from GF diets due to conflicting scientific findings. While a few studies reported potential harm in a small number of CD adults [95,96], other well-documented studies demonstrated the safety of oats for CD children and adults [97,98,99,100]. Cross-contamination with gluten-containing cereals can occur while growing, transporting, and milling oats alongside other grains. Studies on oat contamination with gluten-containing cereals have mainly focused on oats sold in the USA and Europe [101,102,103]. A study in Canada [104] revealed significant gluten contamination in commercial oat samples, with approximately 88% of samples (n = 133) having gluten levels above 20 mg/kg, regardless of oat type. Fermentation plays a crucial role in reducing gluten content in oats. Specific microorganisms produce enzymes that break down gluten proteins into less harmful forms, making the final fermented product safer for individuals with CD or gluten sensitivity [105]. As a result, fermentation offers a promising approach to address gluten-related concerns in oats.

In an intervention study, Aparicio-García and colleagues [12] investigated the safety of a fermented beverage made from sprouted oats (SOFB) using *L. plantarum WCFS1* and its impact on nutritional serum biomarkers and intestinal microbiota composition in adults with CD. The study involved ten celiac adults (aged 22–64 years) adhering to a gluten-free diet for at least two years. They were randomly divided into two groups: the treated group received 200 mL/day of SOFB, and the control group received a gluten-free placebo. The intervention period was six months, during which participants maintained their gluten-free diet. Clinical and immunological markers were monitored at 0, 2, 4, and 6 months. SOFB consumption was well-tolerated and safe, as it did not induce mucosal damage or immunoglobulin A antibodies against tissue transglutaminase (IgA anti-tTG antibody) production. In terms of its effects, SOFB did not lead to weight changes, but it positively impacted cholesterol levels, increasing beneficial gut bacteria like *lactobacilli* and *Ruminococcus* while decreasing inflammatory markers. The results suggest that SOFB could be a promising adjunct in celiac disease treatment, but further research in a larger population is needed.

## 6. Future Perspectives for Fermented Oats

The market currently has only a few fermented oat products available, with European countries (especially Scandinavian countries) and the United States leading in their promotion and production. Bacteria have been more commonly used in developing fermented oats compared to fungi and yeasts. However, research on fermented oats and their health benefits is still in its early stages, mostly relying on limited in vitro, rodent, and human studies. To better understand the health effects of fermented oats, more research is needed, especially in human in vivo models.

Previous studies have shown that fermentation can increase the levels of certain C-type AVAs and phenolic acids in fermented oats compared to unfermented ones. However, the impact of fermentation on other bioactive compounds like steroidal saponins [81,106], triterpenoid saponin [79], and A-type AVAs [80] in oats remains unknown. Further studies are required to investigate these changes and their influence on the health benefits of fermented oats.

While some studies have indicated beneficial effects of fermented oats on conditions such as diabetes, hyperlipidemia, obesity, cancer, and celiac disease, based on in vitro and in vivo experiments, as well as human studies using extracts and fermented oat beverages, further research using whole fermented oats is essential to establish their potential as therapeutic interventions for these health conditions. These comprehensive investigations will help us better understand the therapeutic benefits of fermented oats and their potential role in managing these pathological conditions. Additionally, exploring the individual contributions of probiotics, β-glucan, and phytochemicals in fermented oats, as well as their potential additive or synergistic effects, is essential to maximize their positive impact on human health. Comprehensive investigations are thus warranted to unlock the full potential of fermented oats as a functional and health-promoting food.

## Data Availability

Not applicable.
